# Reproductive consequences of an extra long-term sperm storage organ

**DOI:** 10.1186/s12862-020-01704-6

**Published:** 2020-11-30

**Authors:** Akashdeep Dhillon, Tabashir Chowdhury, Yolanda E. Morbey, Amanda J. Moehring

**Affiliations:** Department of Biology, Western University, London, ON N6A 5B7 Canada

**Keywords:** Sperm storage, Spermathecae, *Drosophila*, Offspring production, Fitness

## Abstract

**Background:**

Sperm storage plays a key role in the reproductive success of many sexually-reproducing organisms, and the capacity of long-term sperm storage varies across species. While there are theoretical explanations for why such variation exists, to date there are no controlled empirical tests of the reproductive consequences of additional long-term sperm storage. While Dipterans ancestrally have three long-term sperm organs, known as the spermathecae, *Drosophila* contain only two.

**Results:**

We identified a candidate gene, which we call *spermathreecae* (*sp3*), in which a disruption cause the development of three functional spermathecae rather than the usual two in *Drosophila*. We used this disruption to test the reproductive consequences of having an additional long-term sperm storage organ. Compared to females with two spermathecae, females with three spermathecae store a greater total number of sperm and can produce offspring a greater length of time. However, they did not produce a greater total number of offspring.

**Conclusions:**

Thus, additional long-term sperm storage in insects may increase female fitness through extending the range of conditions where she produces offspring, or through increasing the quality of offspring via enhanced local sperm competition at fertilization.

## Background

Reproductive success in sexually-reproducing organisms with internal fertilization systems relies, in part, on the separation of mating and fertilization. The processes of recruitment, maintenance, and utilization of sperm is enhanced by specialized organs within the female reproductive tract, referred to as sperm-storage organs. These organs are present in most animal species, including bats [1, 2], birds [3, 4], reptiles [5], molluscs [6], arachnids [7], and insects [8]. Sperm storage can limit costs associated with multiple matings, increase sire choice, ensure fertilization, and align reproductive demands with resource availability [9, 10]. Post-copulatory sexual selection has influenced the appearance, abundance, and function of these organs [11, 12], and there is some evidence that long-term storage organs experience stronger positive selection than short-term sperm storage organs, based on the nucleotide divergence of genes involved in storage organ development [13].

Within insect species, short-term sperm storage occurs in the seminal receptacle, while long-term sperm storage capacity is provided by the spermathecae (reviewed in [10, 12]). Only a portion of male ejaculate is stored within a female, and long-term sperm viability is partially due to spermathecal morphology [9]. In Diptera, spermathecae abundance can vary from complete absence up to four, with three being the most common and presumed ancestral state of the Order [14]. One theory as to why the number of spermathecae varies among species is that spermathecae number depends on the requirements of sperm longevity, utility, and seminal protein influence on ovulation, and thus is negatively correlated with re-mating frequency [12, 15–17]. The assumption is that the greater the number of spermathecae, the longer a female is able to produce offspring, or the more offspring are produced, following a single mating. However, there is likely an energetic cost to the production and maintenance of sperm storage organs [12], and thus there is a selective balance between the benefits of additional storage organs and the costs.

Female *Drosophila melanogaster* contain two spermathecae, and sperm from these long-term storage organs are used from approximately five days to three weeks post-mating [10, 18, 19]. Though genetic pathways involved in spermathecal development in *Drosophila* have yet to be fully mapped, their formation results from the evagination of the genital imaginal discs [20, 21]. Approximately four hours after puparium formation, compartments begin to persist with mediation by signal transduction pathway genes, such as *hedgehog*, *decapentaplegic*, and *wingless* [20, 22, 23]. Families of guanine nucleotide exchange factors and epithelial E-cadherin junction formation pathways are also involved in spermathecae development, and disruptions in these pathways can cause additional spermathecae to develop [24].

Here, we identify a novel gene disruption that causes three spermathecae to develop, rather than the usual two, in *D. melanogaster*. We then show that the extra spermathecae appears to be functional. This allowed us to assess the consequences of additional long-term sperm storage on reproductive efficiency in two ways: (1) sperm utilization, as measured by sperm loss from each spermatheca over time, and (2) offspring production, as measured by the number of adult flies produced over time.

## Methods

### Fly husbandry

**Fig. 1:**
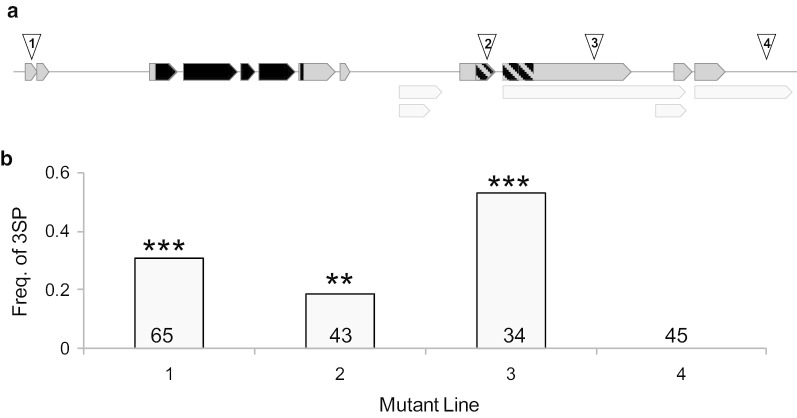
Tests of gene disruptions. **a** Exons of gene *CG7956* (a.k.a. *spermathreecae*) are shown as arrows, with light grey arrows denoting alternative splice variants of exons. The black portions of the exons represent the region coding for the protein’s SAC domain, while the hashed exons represent the region coding for the Inositol phosphatase domain. Insertion locations, denoted by numbered triangles, for the original experimental line (3: *P{GawB}1471*) and the three additional lines (1: *Mi{ET1}CG7956*^*MB05039*^, 2: *Mi{Mic}CG7956*^*MI01858*^, and 4: *PBac{IT.GAL4}CG7956*^*0347-G4*^). Insertions 1, 2, and 3 are in the same orientation as the gene, while insertion 4 is of unknown orientation. **b** Proportion of females with three spermathecae, with the sample size listed on each bar. Significant differences between observed frequency of three spermathecae and expected value within lines are indicated as: **P < 0.01, ***P < 0.001

*Drosophila melanogaster* fly stocks were acquired from Bloomington *Drosophila* Stock Center unless otherwise noted. Scoring disruptions in gene *CG7956* (Fig. 1): stock #9465 (*w*^*1118*^*; P{GawB}1471*), #24847 (*w*^*1118*^*; Mi{ET1}CG7956*^*MB05039*^), #34454 (*y*^*1*^w^*^; Mi{Mic}CG7956^*MI01858*^), and #64712 (*w*^*1118*^*; PBac{IT.GAL4}CG7956*^*0347-G4*^). Control stocks for rate of three spermathecae in other *P*-element disruption lines: stock #30815 (w^*8^*; P{w*^+*mW.hs*^ = *GawB}fru*^*NP0021*^) and #30815 (w^*^; P{w^+*mW.hs*^ = *GawB}121Y*). A stock with GFP-tagged sperm heads (*w; P{w8, ProtA-EGFP, w* +*}19B(3)*, henceforth called GFP-sperm) was provided by Dr. John Belote. All stocks were maintained at 24 °C and ~ 75% relative humidity on a 14:10 h light:dark cycle. Flies were grown on standard cornmeal-agarose-corn syrup food medium (Bloomington *Drosophila* Stock Center recipe).

### Frequency of three spermathecae

**Fig. 2:**
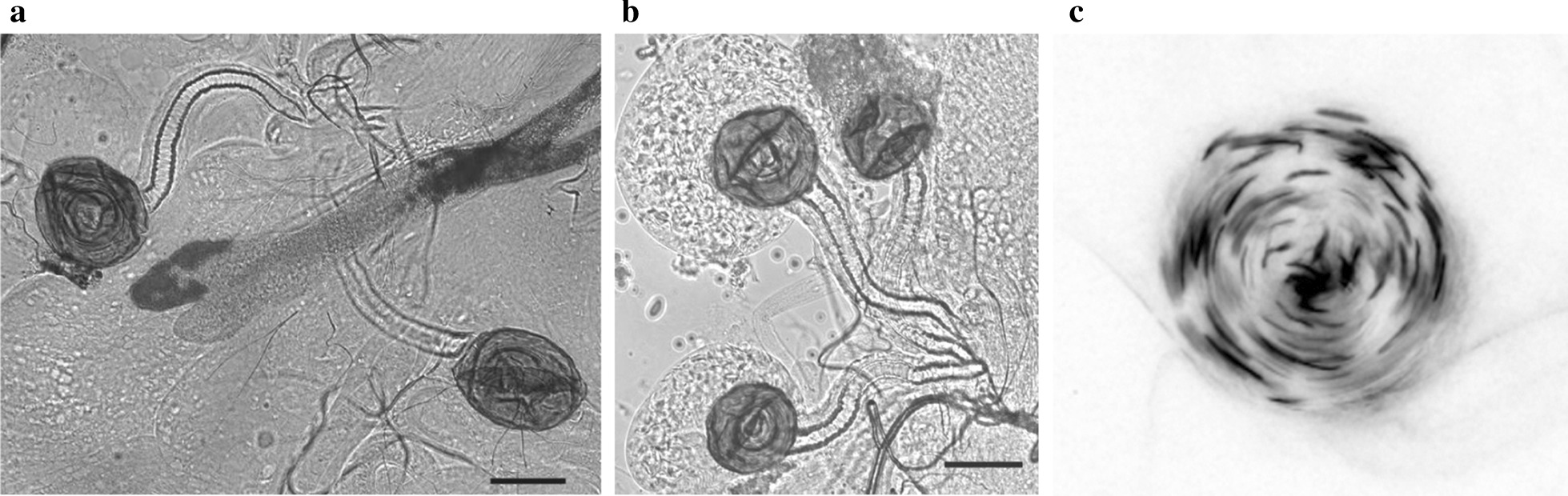
Spermathecae phenotypes. Phase contrast images of **a** two and **b** three spermathecae phenotypes. Scale bar is 50 μm. **c** Fluorescent microscopy image of GFP-labeled sperm within a single spermathecae; image color is inverted to enhance visibility. Note that this is a single image from a z-stack, and thus not all sperm are in focus in this image

The increased number of spermathecae per female was initially observed by chance in *P{GawB}1471* females; the insertion site of this disruption within the genome was initially unknown (see Inverse PCR, below). Females were placed in a − 20 °C freezer for 10 min to slow their movement, decapitated, and placed in a drop of testes buffer (aqueous solution: 183 mM KCl, 47 mM NaCl, 10 mM Tris–HCl). Micro-dissection tweezers were used to remove the reproductive organs, as a single unit, from the body. Once removed, the number of spermatheca present was recorded (Fig. 2a, b). Classification of two and three spermathecae flies include flies with spermathecae that appeared phenotypically normal and did not contain malformed spermathecae. Females with spermathecae that were malformed or conjoined at the head, a rare occurrence (< 5%), were discarded. Flies with three spermathecae generally have two spermathecae that share a partly separated spermathecal duct, and these are recorded as containing three spermathecae. A minimum of 30 females for each stock were scored. Control *P-*element insertion lines *P{GawB}fru*^*NP0021*^ and *P{GawB}121Y* were also scored for the incidence of three spermathecae in order to determine the ‘expected’ incidence of three spermathecae. As the results from these two control strains were identical, they were pooled in subsequent statistical analyses. A Fisher’s exact test was utilized to test whether the observed frequency of three spermathecae was greater than expected based on the control lines.

### Matings

A single virgin GFP-sperm male and virgin *P{GawB}1471* female, both aged 4 days, were aspirated as single pairs into a 30 mL food vial containing standard food media and plugged with cotton that was pushed down to give ~ 1 cm maximum height of air space to increase mating likelihood. Flies were observed to ensure one complete mating had occurred, indicated by ~ 20 min-copulation time prior to male and female decoupling. After decoupling, males were removed within ~ 1 min via aspiration. For sperm utilization and reproductive output assays, females remained in the vials. A maximum of 20 individual mating pairs were set up at one time.

### Sperm utilization assay

Reproductive tracts of mated females were dissected and scored at one of five timepoints after mating occurred: 1 day (24 h), 5 days, 10 days, 15 days, or 20 days. For the latter three groups, females were tipped into new individual food vials every five days. Reproductive tracts were dissected using the same protocol used to assess spermathecae number (see above). The use of GFP-sperm males in mating assays facilitated counting the number of sperm within the sperm-storage organs (Fig. 2c). Sperm within the storage organs was visualized as a z-stack using a Nikon Eclipse Ci fluorescent microscope, with images of each spermatheca taken individually using a Nikon DS-Fi1 camera and NIS-Elements D software. Image file names were then "blinded" so that the scorer did not know the age or spermathecae number of the female. Sperm number within each spermatheca was scored using ImageJ software. Utilizing a two-tailed unequal variance t-test, sperm number per spermatheca was compared between biological groups (2 vs. 3 spermathecae). Based on this, we expected three-spermathecae females to have more total sperm.

Initial exploration of the sperm count data followed [25]. Zero-inflation (15% of data) and overdispersion warranted zero-inflated generalized linear models (zGLM) with negative binomial error distributions and the log link function for the count component of the model. The zero component of the model assumes a binomial error distribution and logit link function. For the total sperm count, we implemented this analysis using function zeroinfl in package pscl [26, 27] in R v3.5.1 [28]. Sperm counts and zeros were modelled as functions of days post copulation (considered as a continuous covariate in all analyses) and the factor group (two vs. three spermathecae). Model simplification was done by comparing nested models with likelihood ratio tests. We also tested for day by group interactions with likelihood ratio tests. For the sperm count per spermathecae data, we needed to account for multiple measurements per female. Thus, we implemented a zero-inflated generalized linear mixed model (zGLMM) using function glmmTMB in package glmmTMB [29]. The fixed effects were the same as those used in the final zGLM of the total sperm count data, and random intercepts were specified for females. For all models, coefficients (β ± 1 S.E) are presented.

### Reproductive output assay

Offspring output data were collected from females used in the 20-day sperm utilization assay, above. Females were tipped into new food vials every 5 days; offspring produced from each of those vials was scored. The vials therefore represent the offspring production of days 0–5, 5.5–10, 10.5–15, and 15.5–20 days after mating. Twenty days after the female was removed from each vial, offspring within the vial were manually counted twice, with the second count for verification purposes.

Offspring count data were collected in a repeated-measures design, showed zero inflation (29% of the data), and showed overdispersion. This warranted the use of a zGLMM with a negative binomial error distribution to compare offspring production between groups and across days post copulation, while accounting for random intercepts for females. Thus, function glmmTMB was used. Choice of predictors for the offspring count and zero components of the model was based on likelihood ratio tests of nested models.

To complement the analyses of offspring production, we compared the timing of egg production between females with two and three spermathecae using the function coxph in the package ‘survival’ [30]. We used this function to fit a proportional hazard model with group as a factor. Dependence within females was accounted for by using the cluster option.

### Inverse PCR

The *P-*element insertion location in *P{GawB}1471* was identified by isolation and sequencing of flanking regions. DNeasy Blood & Tissue Kit from QIAGEN was used for DNA isolation following standard kit protocol, with the following modifications. Separate sequential homogenization steps with ATL buffer were combined to compensate for the volume of fly bodies used, thus addition of 100 µl followed by 80 µl was substituted by a singular addition of 180 µl of ATL buffer. AE Buffer, used for elution of DNA through column, was substituted with DNase free distilled water.

DNA concentration, determined using a Nanophotometer P300, was used to calculate appropriate volumes of samples for the restriction enzyme digest. The protocol for iPCR was designed based on that from the Berkeley *Drosophila* Genome Project website (accessed August, 2017: https://www.fruitfly.org/about/methods/inverse.pcr.html). Reaction ratio was scaled down by 1:5 as six flies were used instead of 50. PCR was performed using the appropriate 3′ or 5′ forward and reverse *P{GawB}*primers based on [31], as follows: *pGawB-5*′*F* GAGGATGACATGTCGGATGG, *pGawB-5*′*R* GTCCGCACACAACCTTTCC, *pGawB-3*′*F* CGGGACCACCTTATGTTATTTC, *pGawB-3*′*R* CTGAGTGAGACAGCGATATG. The PCR reaction was as follows: 1 cycle 95 °C 3 min; 30 cycles 95 °C 30 s/59 °C 30 s/72 °C 45 s; 1 cycle 72 °C 1 min.

PCR products were run on a 1.5% agarose gel. DNA gel extraction of bands was performed using a QiaQuick Gel Extraction Kit following standard kit protocol. Nucleic acid concentration was determined on a Nanophotometer P300 prior to sequencing at Robarts Research Institute. Upon return of sequence data, chromatograms were assessed to ensure clean and accurate sequence data utilizing Geneious version (11.0.4) (https://www.geneious.com, [32]). Sequences were then aligned to the *D. melanogaster* genome database using NCBI’s basic local alignment search tool (BLAST).

### RT-PCR

The qualitative amount of transcript present for *CG7956* in disruption line *P{GawB}1471* was determined using RT-PCR. Adult females aged 5–7 days were dissected in 1 × PBS buffer and transferred to separate micro-centrifuge tubes based on the number spermathecae present (2 vs. 3). Total RNA was extracted from 10 females with either two or three spermathecae using TRIzol and a Purelink RNA purification kit (Thermofisher Cat# A33254). RNA was quantified using a Nanophotometer P300 (Implen, Inc.) and 2 µg of total RNA was used for cDNA synthesis using Maxima First Strand cDNA Synthesis Kit with DsDNAase (Thermofisher, Cat# K1671). RT-PCR was performed to determine the presence of *CG7956* transcripts using the following forward and reverse primers: IPP-Fwd: TCTCGAAATTGGGACAGACC; IPP-Rev: ATCTCCACATCCGAGTCCAC. *RpL32* was used as a control to compare gene expression levels using the following primers: Rpl32-Fwd: GACGCTTCAAGGGACAGTATCTG; Rpl32-Rev: AAACGCGGTTCTGCATGAG. These primers amplify sequence within the 10^th^, and largest, exon, just upstream of the *P*-element insertion site. We chose this upstream location as there are splice variants downstream of the insertion site, and we wanted to capture the expression of all transcripts. The RT-PCR thermocycler protocol was as follows: 95 °C 3 m; 30 cycles 95 °C 30 s/57 °C 30 s/72 °C 30 s; 72 °C 5 m.

### Testing of additional lines for three spermathecae

Females from three additional disruptions in gene *CG7956* (*Mi{ET1}CG7956*^*MB05039*^, *Mi{Mic}CG7956*^*MI01858*^, and *PBac{IT.GAL4}CG7956*^*0347-*G4^) were scored for the frequency of three spermathecae using the same protocol detailed above for *P{GawB}1471* females*.*

## Results

**Fig. 3:**
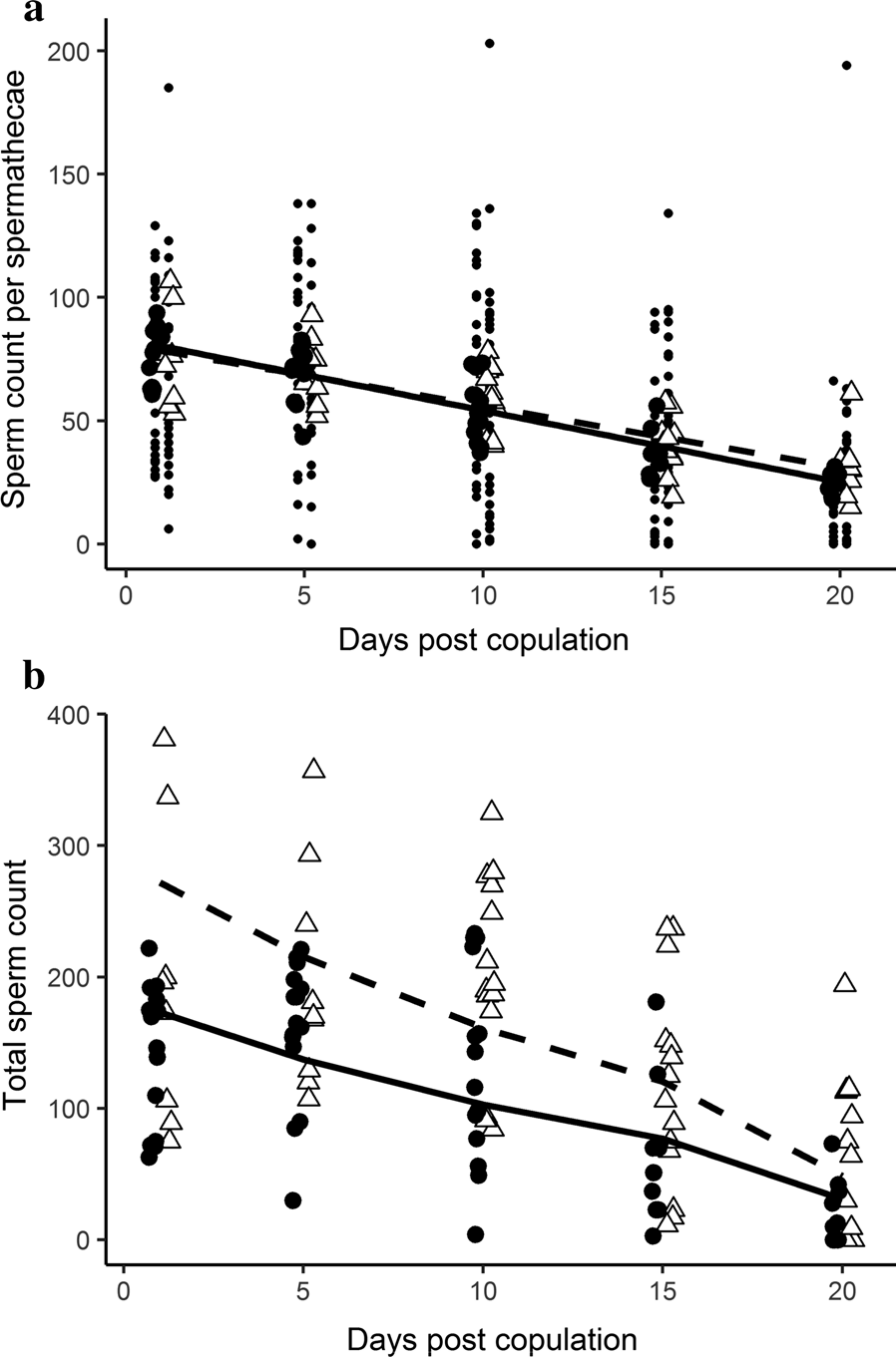
Sperm counts for flies with two (large filled circles, solid line) or three (large open triangles, dashed line) spermathecae. The data are jittered and dodged to better display overlapping values. **a** Sperm count per spermathecae versus days post copulation. The small circles show the raw data (2–3 values per female per day), whereas the large symbols show the predicted values for each female. The lines represent the least square regression lines for the predicted (conditional on the zero model) values. **b** Total sperm count versus days post copulation. The lines represent the predicted values from the zero-inflated generalized linear model. The data are jittered and dodged to better display overlapping values

Compared to the two control *P*-element insertion strains, which had no incidence of three spermathecae (*P{GawB}fru*^*NP0021*^: 0/20, *P{GawB}121Y*: 0/26), a significant proportion of females bearing the insertion *P{GawB}1471* had three spermathecae (freq. = 0.529; n = 34; *P* < 0.00001; Fig. 1). While we did not quantify sperm motility, we note that motile sperm was consistently observed in all three spermathecae. To assess sperm utilization between two- and three-spermathecae groups, the number of sperm within each spermathecae was scored at 1-day, 5-day, 10-day, 15-day, and 20-day time points after a single mating (Fig. 3; Additional file 1: Table S1; Additional file 2: Fig. S1). We did not score the number of sperm present within the seminal receptacle since this sperm storage organ is involved in short-term sperm storage rather than long-term sperm storage (reviewed in [10, 12]). The zGLM and zGLMM for sperm count data included the predictors day and group for the count component, and day for the zero component. Thus, groups did not differ in the occurrence of zeros.

There was no significant difference in the number of sperm within each spermathecae for females with two *vs.* three spermathecae (zGLMM, β = 0.06 ± 0.12, z = 0.52, *P* = 0.60) in the model that controlled for a decline in sperm count with days post copulation (β = − 0.06 ± 0.01, z = − 5.8, *P* < 0.0001; Fig. 3a) and an increase in the log odds of zero counts with days post copulation (β = 0.34 ± 0.06, z = 6.0, *P* < 0.0001). This indicated that each one of the three spermathecae stored as much sperm as each of the two spermathecae. As a result, three-spermathecae flies contained significantly more total sperm compared to two-spermathecae flies (zGLM, β = 0.45 ± 0.12, z = 3.7, *P* = 0.0002; Fig. 3b). Flies with three spermathecae had 1.57 (e^0.45^) times the total sperm count as flies with two spermathecae.

**Fig. 4:**
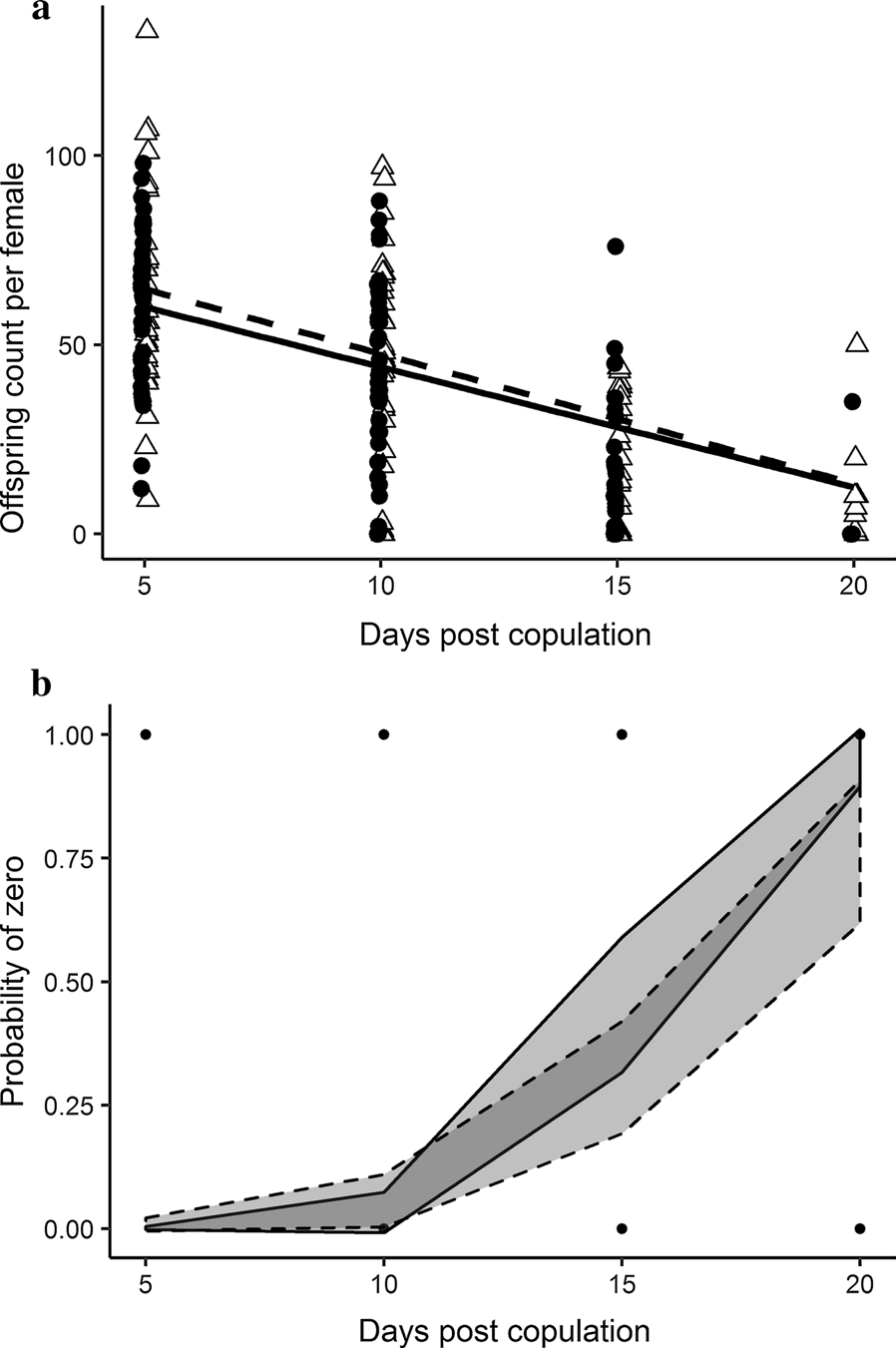
Offspring production over time. **a** Offspring production versus days post copulation for flies with two (filled circles, solid line) or three (open triangles, dashed line) spermathecae. The lines represent the least square regression lines for the predicted values for each female, conditional on the zero model (not shown), from the zero-inflated generalized linear mixed model. The data are jittered and dodged to better display overlapping values. **b** Model of zero offspring production versus days post copulation for flies with two (solid line) or three (dashed line) spermathecae. The shaded areas show 95% confidence intervals, with the darker shading simply due to overlap of the confidence intervals for the two groups. The solid circles show the data and represent many overlapping data points

To assess whether there was increased reproductive efficiency for three-spermathecae females, we scored how many offspring a female produced after a single mating then scored her for the number of spermathecae she contained (Additional file 1: Table S2; Additional file 2: Fig. S2). The zGLMM for offspring counts included the predictors days post copulation and group for the count component, and days post copulation, group, and the day by group interaction for the zero component. Offspring production declined with days post copulation (zGLMM, β = − 0.09 ± 0.01, z = − 9.3, *P* < 0.0001) but did not differ between flies with 2 versus 3 spermathecae (β = 0.08 ± 0.08, z = 0.9, *P* = 0.35; Fig. 4a). The log odds of zero egg production increased with days post copulation (β = 0.64 ± 0.12, z = 5.5, *P* < 0.0001), but overall did not depend on group (β = 2.99 ± 2.12, z = 1.4, *P* = 0.16). Although non-significant, the interaction suggested that the increase in zeros was steeper for females with two spermathecae (β = − 0.24 ± 0.14, z = − 1.8, *P* = 0.078; Fig. 4b). The incidence of zeros most clearly differed between groups on days 15–20 post copulation.

The survival analysis indicated similar offspring production schedules between females with two and three spermathecae (β = − 0.15 + 0.10, z = − 1.5, *P* = 0.13). The direction of the effect and the relatively low *P*-value hint at earlier cessation of offspring production among females with two spermathecae compared to those with three spermathecae. The hazard for producing offspring in females with three spermathecae was 0.86 (e^−0.15^_;_ 95% CI 0.71, 1.05) that of females with two spermathecae.

Using inverse PCR followed by sequencing, we identified the *P{GawB}1471* insertion location as being within the gene *CG7956.* Unsurprisingly, this disruption did not affect qualitative levels of *CG7956* transcription (Supplementary Figure S3), as the insertion location within an exon likely exerts its effect via disruption in protein functionality rather than disruption of transcription. Two of the three additional disruption lines that we tested within this gene (Fig. 1) also had a higher frequency of the three-spermathecae phenotype compared to the control lines: *Mi{ET1}CG7956*^*MB05039*^ (freq. = 0.308; *P* < 0.00001) and *Mi{Mic}CG7956*^*MI01858*^ (freq. = 0.186; *P* = 0.0021). No atypical spermathecal development occurred due to insertion *PBac{IT.GAL4}CG7956*^*0347-G4*^ (freq. = 0; *P* = 1.0), which may not disrupt the gene as it has an unknown orientation within the gene’s 3′ untranslated region (Fig. 1). We combined the four independent disruption tests of *CG7956* into a meta-analysis, which was also statistically significant (Fisher’s method: *P* < 10^–9^), supporting the conclusion that this locus gives rise to the three-spermathecae phenotype.

## Discussion

Here we identified that disruptions in the candidate gene *CG7956* cause females to frequently produce three spermathecae, rather than the typical two spermathecae in *Drosophila melanogaster*. This effect was confirmed with three independent insertions, in locations spanning 6 kb, and there are no other genes in immediate proximity, making it highly unlikely that the effect on spermathecal development is due to the insertions’ effect on another locus. Due to its effect in inducing three spermathecae, we rename this gene *spermathreecae* (*sp3*). We note that this gene’s effect remains to be confirmed via transgenics. This gene has its highest expression levels in the Malpighian tubules, which are part of the renal system, but also has enriched expression in the spermathecae [33]. While it is possible for this locus to affect reproduction via other tissues, the most likely location of the effect on spermathecae number is via its expression within the spermathecae. The SP3 protein is predicted to contain a SAC domain, which acts to hydrolyse phosphate from inositol, and other genes with this same function are known to be involved in tissue morphogenesis [34, 35]. The phosphate hydrolysis function of SP3 likely acts to suppress the development of the third ancestral spermathecae, but confirmation of this role, and the pathway through which it acts, remains to be determined. Since there can be an interplay between the protein products of the spermathecae and the motility of sperm within the short-term sperm storage organ, the seminal receptacle (e.g. [36]), the potential effects of *sp3* on this short-term sperm storage organ should also be examined in future studies.

We used a disruption in *sp3*, and the resulting development of a third spermatheca, as a tool to test the effect of additional long-term sperm storage organs on reproductive output. As we were not able to test the fertilization ability of sperm from each of the spermathecae individually, it is possible that the third spermatheca is not a functional long-term sperm storage organ. However, several lines of evidence indicate that the additional spermatheca is functional. First, there was the same number of sperm initially stored within each of three spermathecae as in each of two spermathecae. Second, sperm was released from each of three spermathecae at the same rate as it is from each of two spermathecae. Lastly, the sperm can be motile within all three spermathecae.

Females that have a greater capacity to store sperm are predicted to have a selective advantage when mates are difficult to find, allowing them to produce more offspring, or produce offspring for longer, after a single mating [12, 15–17]. We found that females with three spermathecae did not produce a greater number of offspring than females with two spermathecae, but they were able to produce offspring longer. Thus, counter to prediction, additional sperm storage organs do not increase the total number of offspring that are produced, at least not in *D. melanogaster*, but they do allow a female to produce offspring for a slightly longer period of time. The absence of greater offspring production may be due to two of the three spermathecae sharing a single terminal duct in many species (e.g. [37]), including in many of the induced three-spermathecae females we scored here, a potential influence that warrants further exploration.

There are several key additional points that stem from our data. Females with three spermathecae had a significantly greater total number of sperm within their long-term sperm storage organs at later time points than females with only two spermathecae. Since these females did not produce more offspring, a larger number of stored sperm did not result in a greater number of offspring in this species. This makes it likely that the reason *D. melanogaster* males transfer more sperm to mated females than virgin females [38] is to increase displacement of rival sperm from the storage organs [39] rather than to increase the total number of sperm within the storage organs. Thus, two spermathecae appear to store as much sperm as is necessary for a *D. melanogaster* female to fertilize all eggs, and additional sperm within the ejaculate does not appear to increase offspring production, but may increase a male’s competitive share of the female’s sperm storage capacity.

Although it does not result in more offspring, additional sperm within the female long-term sperm storage organs may also be beneficial in terms of female fitness. Since sperm is removed at the same rate from each of the spermathecae, females with three spermathecae are depositing more sperm on each egg than females with only two spermathecae. With only a single mating, this can potentially result in higher ‘local sperm competition’, whereby sperm from the same male competes for fertilization of an egg [40, 41]. Three-spermathecae females could have more sperm competing for fertilization of each egg, and thus there may be an advantage of higher quality rather than quantity of offspring in Dipterans. Further, some sperm are able to survive longer, and females with three spermathecae, by sheer numbers, are more likely to have these sperm available at later time points, which allows them to produce offspring slightly longer than two-spermathecae females. This benefit may underlie the presence of three- and four-spermathecae phenotypes among Dipteran species.

## Supplementary information

**Additional file 1: Table S1.** Number of sperm per spermathecae. If no number is listed for the "SP3" column, that female only had two spermathecae. "Day" is the number of days after fertilization at which the spermathecae's sperm numbers were scored. "Female #" is a designated number for each female scored. "SP1-3" are the three separate spermathecae, with a number (1-3) randomly assigned to each spermathecae. **Table S2.** Number of offspring for each time interval (days 0-5, 5.5-10, 10.5-15, and 15.5-20) for female with two vs. three spermathecae (SP#). Females that were not scored for the last time interval have that value left blank. Note that "Female #" is a designated number for each female scored and does not represent the same numbered female as in Table S1.

**Additional file 2: Fig S1.** Individual values for number of sperm per spermathecae forfemales with two (2SP) or three (3SP) spermathecae at each of five time points. **Fig. S2.** Individual values for number of offspring per female forfemales with two (2SP) or three (3SP) spermathecae for each of five time intervals. **Fig. S3.** RT-PCR results for gene *CG7956* (a.k.a. *spermathreecae*) in females from line *P{GawB}1471* that have two (2sp) vs. 3 spermathecae (3sp) compared to wild-type (WT) females. Expression levels of *RpL32* were used as a control for reaction efficiency. NTC = no template control.

## Data Availability

All data generated or analysed during this study are included in this published article and its supplementary information files.

## Abbreviation

sp^3^: Spermathreecae
