# Supplementary material for: Reproductive consequences of an extra long-term sperm storage organ
Source: BMC Evol Biol. 2020 Nov 30;20:159. doi: 10.1186/s12862-020-01704-6 (PMC7706275; doi:10.1186/s12862-020-01704-6)
Supplement: Supplementary file 2 — Additional file 2: Fig S1. Individual values for number of sperm per spermathecae forfemales with two (2SP) or three (3SP) spermathecae at each of five time points. Fig. S2. Individual values for number of offspring per female forfemales with two (2SP) or three (3SP) spermathecae for each of five time intervals. Fig. S3. RT-PCR results for gene CG7956 (a.k.a. spermathreecae) in females from line P{GawB}1471 that have two (2sp) vs. 3 spermathecae (3sp) compared to wild-type (WT) females. Expression levels of RpL32 were used as a control for reaction efficiency. NTC = no template control. [file 12862_2020_1704_MOESM2_ESM.docx]

**Supplementary Figure S1.** Individual values for number of sperm per spermathecae for females with two (2SP) or three (3SP) spermathecae at each of five time points.

1 1 5 5 10 10 15 15 20 20

**Supplementary Figure S2.** Individual values for number of offspring per female for females with two (2SP) or three (3SP) spermathecae for each of five time intervals.

0 - 5 5.5 - 10 10.5 - 15 15.5 - 20

**Supplementary figure S3.** RT-PCR results for gene *CG7956* (a.k.a. *spermathreecae*) in females from line *P{GawB}1471* that have two (2sp) *vs.* 3 spermathecae (3sp) compared to wild-type (WT) females. Expression levels of *RpL32* were used as a control for reaction efficiency. NTC = no template control.


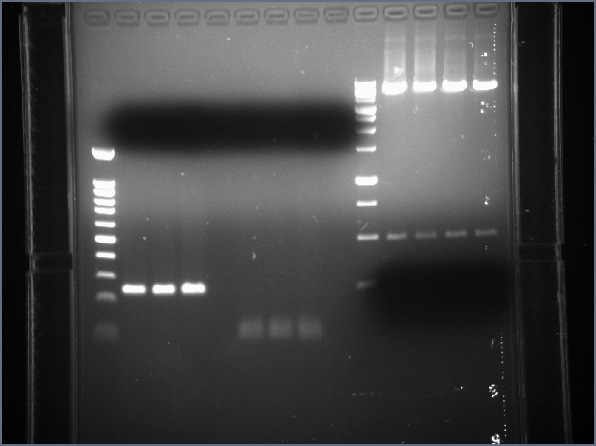


2sp 3sp WT NTC

*CG7956*

2sp 3sp WT NTC

*RpL32*

100bp

ladder
